# The Effects of Cigarette Smoke Extract on Ovulation, Oocyte Morphology and Ovarian Gene Expression in Mice

**DOI:** 10.1371/journal.pone.0095945

**Published:** 2014-04-28

**Authors:** Zixin Mai, Ming Lei, Bolan Yu, Hongzi Du, Jianqiao Liu

**Affiliations:** Department of Reproductive Medicine Center, Key Laboratory for Reproductive Medicine of Guangdong Province, Third Affiliated Hospital of Guangzhou Medical University, Guangzhou, China; Medical Faculty, Otto-von-Guericke University Magdeburg, Medical Faculty, Germany

## Abstract

Cigarette smoking can harm fertility, but the existing research has targeted primarily on ovarian follicles, embryos or sex hormone. In this study, we tested cigarette smoke extract on ovulation, oocyte morphology and ovarian gene expression associated with inhibition of oxidative stress using C57BL/6 mice. Mice in the experimental group were administered a cigarette smoke extract (CSE) solution (2 mg/ml) orally daily, while the blank control group was given dimethylsulfoxide (DMSO). A positive control group (menadione) was used that received an intraperitoneal injection of 15 mg/kg menadione in oil solution daily. We found that the CSE group manifested a reduced diameter of zona pellucida-free oocyte (ZP-free OD) and a morphologically misshapen first polar body (PB). Our results suggest that CSE exposure is associated with a shrink size and poor quality of oocytes. Quitting smoking is a wise choice to ensure good fertility.

## Introduction

The prevalence of smoking among women of reproductive age has increased worldwide over the last several years [1], [2]. There is evidence that 90% of smokers start this behavior during adolescence [3], and young women constitute the fastest-growing population of smokers [4]. It has been reported that cigarette smoking harms the reproductive system in many aspects [5], [6]. Cigarette smoke contains polycyclic aromatic hydrocarbons (PAHs) [e.g. benzo(a)pyrene (B[a]P)], aromatic amine, N-nitroso compounds, heavy metals [e.g. cadmium (Cd)], and so forth [7], [8]. Some studies have indicated a significantly higher level of smoking toxicants in reproductive tissues or fluids than in serum [9], [10], which suggested that the toxicants accumulated in reproductive organs [11]. And smoking may cause deleterious effects on ovary and abnormal sex steroid hormone concentrations [12], [13]. The adverse effects of cigarette smoking on fertility and their relation to premature ovarian failure have also been demonstrated [14]. Smoking is correlated to higher infertility risk [5], [15], lower fecundity rate [16], [17], lower *in-vitro* fertilization (IVF) success rates [18]–[20] and increased rate of spontaneous abortion [21]–[24].

Many of the studies investigating the mechanisms underlying cigarette smoking and fertility concerned the effects of the inherent toxicant molecules on follicles: e.g. B[a]P, a component of cigarette smoke, caused few of ovarian follicles [25], PAHs reduced numbers of primordial and primary follicles in rats and mice [26], and the cigarette toxicants stimulated reproductive organs in a way that was harmful to ovarian follicles, causing follicle depletion [27]–[29] and inhibition of follicle growth [13]. Huang focused much more on the embryos and found that cigarette smoke induces compromises to embryo development in vivo [11]. In addition, there are several studies about the effect of smoke on oocytes, such as thicker ZP, higher incidences of chromosomal abnormalities [30], [31] and shrink size [4]. Also, the number of retrived oocyte had been studied, without consistent opinion [32], [33]. However, there were few studies published simultaneously regarding ovulation number, oocyte morphology and ovarian gene expression to reflect the effect of cigarette smoke on oocyte or ovary before fertilization.

Besides, production of reactive oxygen species (ROS), which include superoxide anion [O_2_] and hydrogen peroxide [H_2_O_2_], is a physiological process and occurs in the cell mainly during the mitochondrial energy metabolism. O_2_ is transformed into a more stable ROS, H_2_O_2_ [34]. When H_2_O_2_ concentrations in the cytoplasm reach above the physiological threshold, it can be removed by cytosolic antioxidant systems of the cell. These antioxidant defense mechanisms may include both enzymatic such as catalase, glutathione peroxidase (GPx) [35], and superoxide dismutase (SOD) [36]. Oxidative stress reflects an imbalance between production of ROS and cellular antioxidant defense mechanisms [37], which may have serious consequences, for instance, enzymatic inactivation, DNA fragmentation, and irreversible damage of mitochondrial DNA, membrane lipids, and proteins, resulting in mitochondrial dysfunction and ultimately cell death [38], [39]. It has been found that the initiation of apoptotic cell death in ovarian follicles and granulosa cells by various stimuli is due to increased ROS [40]. SOD2 encodes the mitochondrial isoform of SOD and detoxifies ROS [41]. Heme oxygenase-1(HMOX1) can catalyze a biochemical reaction and the products of the HMOX reaction have an important effect, such as antioxidation [42]. Nuclear factor erythroid 2-related factor 2 (NRF2) regulates transcription of genes that encode enzymes important for protection against ROS [43]. Glutathione-s-transferases (GSTs) can catalyze the conjunction between intermediate metabolites of xenobiotic metabolism and glutathione (GSH), achieving detoxification [44]. As one of the GSTs, glutathione-S-transferase P1 (GSTP1) enzyme selectively detoxifies the carcinogenic epoxide of B[a]P, a highly carcinogenic metabolite of PAHs [45]. Glutathione-s-transferase Mu 1 (GSTM1), Mu 2 (GSTM2) and glutathione-s-transferase Alpha3 (GSTA3) also belong to the GSTs. One of the rate-limiting enzymes of GSH synthesis, glutamate cysteine ligase (GCL), is composed of modifier (GCLM) and catalytic subunits (GCLC) [46], which effect the detoxification directly.

## Materials and Methods

### Ethic statement

The study was approved by the Ethics Committee of the Third Affiliated Hospital of Guangzhou Medical University, and all animal studies were performed under an institutionally approved protocol according to the guidelines and the criteria from the committee.

### Experimental animal preparation

Twenty-four four-week-old female C57BL/6 mice were purchased from the Laboratory Animal Centre of Zhongshan School of Medicine of Sun Yat-sen University. The number of the mice refers to the study from Sobinoff [47]. C57BL/6 mice has many advantages such as strain stability and easy-to-bred, and the sequencing of their genome has completed. So, this strain is always considered as a standard inbred strain, widely used in the genetics, immunology and pathology study. The mice were randomly divided into three groups, 8 for each, and maintained on a controlled light cycle schedule of 12∶12 h (light/dark) at 25°C with food *ad libitum*.

### Preparation of cigarette smoke extract and menadione oil solution

We obtained cigarette smoke extract using the SHZ IIID-type, multi-use recycled water system. Joint the SHZ IIID-type, multi-use recycled water system with a filter flask, which contained 100 ml dimethylsulfoxide (DMSO, Sigma-Aldrich^®^ D2650-100 ML; St. Louis Missouri USA). The filter with cigarette was inserted into the glass tube of the filter flask, and then the cigarette was lighted up under 0.1 MPa vacuum pressure. Changing the cigarette one by one after being burned out, we used 40 cigarettes for per 100 ml DMSO. The concentration was 8.767 g/100 ml DMSO.

Menadione powder (SIGMA M5625-100G) was dissolved in corn oil (Gold Arowana, China) to obtain a concentration of 2.55 mg/ml.

### Animal dosing

We only gave a CSE oral solution to mice daily, and maturation of cumulus oocyte complexes (COCs) was allowed to occur in vivo; this would maintain stable absorbance of the CSE and a stable serum concentration of the inherent toxicants in smoke, unlike the smoke administered method via nose several times daily used by Huang [11], which may cause unstable serum concentration. Additionally, the effect of carbon monoxide was not tested.

The CSE group was only administered a 2 mg/ml CSE solution (with distilled water as solvent) orally daily *ad libitum*, while the control group was given an equal concentration of the DMSO solution (in distilled water) *ad libitum*. Those from CSE received 4.01 ml/d for each, while 5.86 ml/d in control group. The menadione group, as a positive control, was given an ip injection of 15 mg/kg of menadione oil solution daily and water *ad libitum*. The dosage and route of administration for menadione were based on several studies and were chosen with the intention of inducing partial ovotoxicity with minimal cytotoxicity [47],[48]. The procedures mentioned above were administrated for four weeks in four-week-old mice.

All mice were superovulated at eight weeks of age via ip injection of 5 IU of equine chorionic gonadotropin (eCG, Zhengjiang Modern Biotechnology, Tianjin, China) followed by ip administration of 5 IU of human chorionic gonadotropin (hCG, Yantai Northern pharmaceutical Co. Ltd, China) 48 h later.

### Ovary removal and oocyte retrieval

Fourteen hours after hCG injection, all the mice were sacrificed. The COCs were isolated from oviducts followed by granulosa cell digestion with HYASE-10X (Vitrolife; Göteborg Sweden), cultured in G-1 PLUS (Vitrolife; Göteborg Sweden), and then observed microscopically.

Ovaries were surgically removed, placed in the cryopreservation tubes, and stored in the liquid nitrogen.

### Oocyte observation and measurement

**Figure 1:**
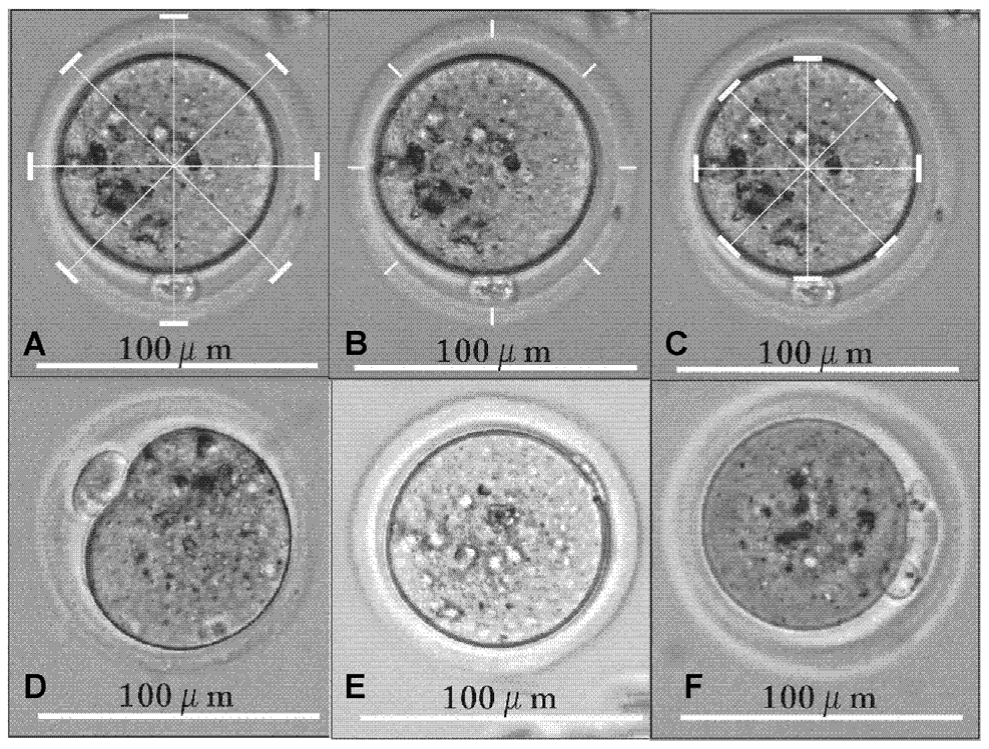
The measurement of OD, ZP and ZP-free OD and different types of first polar body. These microscopy pictures were taken from digital photos using a LEICA inverted microscope (LEICA DM IL LED; Wetzlar, Germany) at ×200 magnification mounted with a camera (LEICA DM6000 B; Wetzlar, Germany). All the measurements were performed with Corel Draw edition 12.0.0525. Figure A. The white lines show the diameter of oocyte (OD). Figure B. The white lines show the thickness of zona pellucida (ZP). Figure C. The white lines show the diameter of ZP-free oocyte (ZP-free OD). Figure D. The first polar body with appropriate size, round shape and smooth surface (ARS-PB). Figure E. The first polar body with small size, strip-like shape and rough surface (SSR-PB). Figure F. Broken PB.

We counted the number of ovulations for every mouse. Measurements of oocyte diameter (OD), ZP thickness and ZP-free OD were taken from digital photos using a LEICA inverted microscope (LEICA DM IL LED; Wetzlar, Germany) at ×200 magnification mounted with a camera (LEICA DM6000 B; Wetzlar, Germany). Diameter was measured at four different locations to obtain a mean, while thickness was measured at eight different locations to obtain a mean (Figure A, B and C in Figure 1). The size of the perivitelline space (PVS) was calculated (PVS = OD–ZP-free OD–ZP thickness×2). All the measurements were performed with Corel Draw edition 12.0.0525. Additionally, we counted the numbers of the first PB in different types as shown in Figure D, E and F in Figure 1.

### RNA extraction from ovaries

Total RNA was extracted from ovarian tissue samples and preserved in TRIzol reagent (Invitrogen; Carlsbad California USA) according to the manufacturer's instructions. Briefly, samples were thawed, placed at room temperature for 10 min, and 0.2 ml of chloroform was added per 1 ml of TRIzol reagent. Sample tubes were securely capped, briefly vortexed, placed at room temperature for 5 min, and then centrifuged at 12 000×g for 15 min at 4°C. The aqueous phase was then transferred to a fresh tube and RNA was precipitated by mixing with 0.5 ml isopropyl alcohol, and placed at −20°C for 30 min. Centrifugation was repeated before removing the supernatant. The RNA was washed twice with 0.5 ml 70% ethanol followed by repeated centrifugation before removing the supernatant. The final RNA pellet was dried and then dissolved in 50 µl of diethyl pyrocarbonate (DEPC)-reated water and placed in a bath at 65°C for 10 min.

### Quantitative real-time reverse-transcription polymerase chain reaction (qRT-PCR)

qRT-PCR was used to assess the expression of ten genes (ACTIN, SOD2, GSTP1, HMOX1, GSTA3, NRF2, GSTM1, GSTM2, GCLM, GCLC) in the ovarian samples. First, cDNA was synthesized using the Reverse Transcription System (Promega A3500; Madison USA) according to the manufacturer's instructions. Briefly, 2 µl of total RNA was reverse transcribed by adding 4 µl of MgCl_2_ (25 mM), 2 µl of RT 10× Buffer, 2 µl of dNTP Mixture, 0.5 µl of Recombinant Rnasin Ribonuclease Inhibitor, 15 u of AMV Reverse Transcriptase (HC; Promega M9004; Madison USA), 0.5 µg of Oligo(dT)15 Primer, and nuclease-free water to a final volume of 20 µl.

The cDNA was prepared in a Mastercycler nexus flat PCR system (Eppendorf; Hamburg Germany) using the following program: 1 cycle at 42°C for 15 min, 1 cycle at 95°C for 5 min and 1 cycle at 4°C for 5 min. At the end of the run, samples were stored at 4°C. The GoTaq® q PCR Master Mix (Promega A6001; Madison USA) was used according to the manufacturer's instructions to perform qPCR analysis of the genes mentioned above and Actin transcript frequency. Briefly, 10 µl of GoTaq qPCR Master Mix, 10 µl of the appropriate primer designed against published mRNA sequences (Table 1) at a concentration of 0.4 µM, and 50-100 ng of cDNA template were added for a final reaction volume of 20 µl. The reaction was performed in the CFX96 Touch™ Real-Time PCR Detection System (Bio-Rad Laboratories #185-5195; Hercules California USA) using the following program: 1 cycle at 95°C for 30 sec, and 40 cycles of 95°C for 5 sec followed by 60°C for 30 sec. After cycling, the temperature was increased starting from 60°C to 95°C at a rate of 0.5°C every 5 sec to generate a melting curve. Samples were amplified in triplicate and a melting curve was completed after each PCR reaction to ensure fluorescence quantification was specific to a single PCR product. The amplification data obtained with qRT-PCR for individual genes was expressed as cycle threshold (Ct), which subtracted the Ct for ACTIN to obtain △Ct (△Ct = Ct [specific gene]-Ct [ACTIN]) followed by 2^−△Ct.^

**Table 1:** Sequences of 10 Relevant mRNA.

Primer name	Gene Bank Accession Number	Sequence(5'to3')
ACTIN-f*	NM_001148849.1	TTGCTGACAGGATGCAGAAG
ACTIN-r*	NM_001148849.1	ACATCTGCTGGAAGGTGGAC
SOD2-f	NM_013671.3	CAGACCTGCCTTACGACTATGG
SOD2-r	NM_013671.3	CTCGGTGGCTTGAGATTGTT
GSTP1-f	NM_013541.1	ATGCCACCATACACCATTGTC
GSTP1-r	NM_013541.1	GGGAGCTGCCCATACAGAC
HMOX1-f	NM_010442.2	AAGCCGAGAATGCTGAGTTC
HMOX1-r	NM_010442.2	GCCGTGTAGATATGGTACAAGGA
GSTA3-f	NM_001077353.1	AAGAATGGAGCCTATCCGGTG
GSTA3-r	NM_001077353.1	AGGTCATCCCGAGTTTTCAGAA
NRF2-f	NM_010902.3	CAGCATGTTACGTGATGAGG
NRF2-r	NM_010902.3	GCTCAGAAAAGGCTCCATCC
GSTM1-f	NM_010358.5	AGCACCCTGGCCTTCTGCACT
GSTM1-r	NM_010358.5	TTCGCAGAAACGGGCTGTGAG
GSTM2-f	NM_008183.3	TACACCATGGGGGACGCTCCT
GSTM2-r	NM_008183.3	TGGCCAACTGTATGCGGGTGT
GCLM-f	NM_008129.4	GCCACCAGATTTGACTGCCTTT
GCLM-r	NM_008129.4	CAGGGATGCTTTCTTGAAGAGCTT
GCLC-f	NM_010295.2	ATGTGGACACCCGATGCAGTATT
GCLC-r	NM_010295.2	TGTCTTGCTTGTAGTCAGGATGGTTT

*f: forword primer. r: reverse primer.

SOD2: superoxide dismutase; GSTP1: glutathione-S-transferase P1; HMOX1: heme oxygenase-1; NRF2: nuclear factor erythroid 2-related factor 2; GSTM1: glutathione S-transferase Mu 1; GSTM2: glutathione S-transferase Mu 2; GSTA3: glutathione S-transferase alpha3; GCLM: glutamate cysteine ligase modifier subunit; GCLC: glutamate cysteine ligase catalytic subunit.

### Statistical analysis

All statistical analyses were performed using SPSS Statistics 17.0. Differences were considered to be significant at P<0.05. The Shapiro-Wilk test was used to determine whether the data were normally distributed (P>0.05). One-way Analysis of variance (ANOVA), the non-parametric Tamhane's T2 test, Kruskal-Wallis, Mann-Whitney U tests or Pearson chi-square test were used in the analysis of gene expression, ovulation number and oocyte morphology.

## Results

### Ovulation quantity and oocyte morphology

The number of oocytes ovulated from the CSE group showed an increase compared with the control group (CSE 21.86±3.70, control 12.00±2.05, P = 0.061, ANOVA, LSD) (Table 2) without statistical significance. One mouse didn't ovulate, which happened in all the three groups (Dataset S1).

**Table 2:** The comparison of ovulation quantity and oocyte morphology of the 3 groups.

Results		Control	CSE	Menadione
Ovulation Quantity^a^ (/mouse mean±SE)		12.00±2.05	21.86±3.70	17.43±4.31
Oocyte Morphology	ZP thickness^a^ (µm mean±SE)	7.59±0.07*	7.43±0.05	7.31±0.06
	ZP-free OD^a^ (µm mean±SE)	76.56±0.12#	76.10±0.13	76.41±0.12
	PVS^b^ (µm median)	9.1328*	9.2818*	10.0474
	OD^c^ (µm median)	100.7656	100.6098	101.1314
	ARS-PB rate (%)	15.19%#	6.62%	7.62%
	SSR-PB rate (%)	7.59%#	24.26%*	13.33%
	Immature oocyte rate (%)	3.57%	3.27%*	9.84%
	The rate of broken PB (%)	2.47%	8.11%	4.55%

ZP: zona pellucida. PVS: perivitelline space. OD: oocyte diameter. ARS-PB: the first polar body with appropriate size, round shape and smooth surface. SSR-PB: the first polar body with small size, strip-like shape and rough surface. CSE: cigarette smoke extract.

a The data was normally distributed (P>0.05, Shapiro-Wilk), with equal variances (P>0.05, ANOVA).

b The data from at least one group was not normally distributed (P<0.05, Shapiro-Wilk test).

c The data was not normally distributed (P<0.05, Shapiro-Wilk test).

*<0.05 versus menadione.

# <0.05 versus CSE.

The ZP thickness non-significantly reduced in the CSE group (CSE 7.43±0.05, control 7.59±0.07, P = 0.082, LSD), while it was significantly decreased in the menadione group (menadione 7.31±0.06, control 7.59±0.07, P = 0.004, LSD) compared with the control group (Table 2, Dataset S2).

There was a significant reduction in ZP-free OD in the CSE group compared with the control group (CSE 76.10±0.13, control 76.56±0.12, P = 0.018, LSD). Also, there was a decrease in the CSE group, compared with the menadione group (CSE 76.10±0.13, menadione 76.41±0.12, P = 0.068, LSD) (Table 2), though with no significance (Dataset S3).

The PVS of the CSE group appeared visually to be larger than in the control group, but not to a statistically significantly extent (CSE 9.2818, control 9.1328, P = 0.379, Mann-Whitney test). However, that of the menadione group was significantly larger than the PVS of either the CSE (menadione 10.0474, CSE 9.2818, P = 0.024, Mann-Whitney test) or control groups (menadione 10.0474, control 9.1328, P = 0.002, Mann-Whitney test) (Table 2, Dataset S4).

The OD in the CSE group appeared smaller than in the control group, but not significantly (CSE 100.6098, control 100.7656, P = 0.642, Mann-Whitney test). In the menadione group there was a contrary change, with the OD visually larger than in control group, but also not significant (menadione 101.1314, control 100.7656, P = 0.192, Mann-Whitney test), with a non-significantly greater size compared with the CSE group (menadione 101.1314, CSE 100.61, P = 0.084, Mann-Whitney test) (Table 2, Dataset S5).

### The morphology of the first PB

The morphologic classification for the first PB is shown in Figure D, E and F in Figure 1. There was a significant reduction in the rate of the first PB with appropriate size, round shape and smooth surface (ARS-PB)(Table 2) in the CSE group compared with the control group (CSE 6.62%, control 15.19%, P = 0.041, Pearson Chi-Square) (Dataset S6).

The rate of the first PB with small size, strip-like shape and rough surface (SSR-PB) is shown in Table 2. There was a significant increase in the incidence of SSR-PB in the CSE group compared with the controls (CSE 24.26%, control 7.59%, P = 0.002, Pearson Chi-Square) and the menadione group (CSE 24.26%, menadione 13.33%, P = 0.034, Pearson Chi-Square) (Dataset S6).

The rate of broken PB in the CSE group was higher than for the menadione group, while the latter was higher than the control group, although these changes were not statistically significant (CSE 8.11%, control 2.47%, menadione 4.55%, P = 0.174, Pearson Chi-Square) (Table 2, Dataset S6).

### Immature oocyte rate

The rate of immature oocyte in the menadione group was non-significantly higher than that in control (control 3.57%, menadione 9.84%, P = 0.089, Pearson Chi-Square) but significantly higher than the CSE group (CSE 3.27%, menadione 9.84%, P = 0.025, Pearson Chi-Square), while there was no significant difference between the latter two groups (control 3.57%, CSE 3.27%, P = 1.000, Pearson Chi-Square Continuous Correction) (Table 2, Dataset S6).

### Ovarian gene expression

The expression of GSTM2 decreased in the CSE group, but not significantly, compared with the control group (CSE 0.3927±0.0897, control 0.5784±0.1210, P = 0.562, ANOVA *post-hoc* Tamhane's T2 test). GSTM2 expression in the menadione group was significantly lower than in the control group (menadione 0.1433±0.0246, control 0.5784±0.1210, P = 0.025, ANOVA *post-hoc* Tamhane's T2 test) and was attenuated compared with the CSE group (menadione 0.1433±0.0246, CSE 0.3927±0.0897, P = 0.093, ANOVA *post-hoc* Tamhane's T2 test) (Table 3) but not to a statistically significant extent (Dataset S9).

**Table 3:** The comparison of gene expression of the 3 groups.

Gene Expression Result	Control	CSE	Menadione
ACTIN	1	1	1
GSTM1^a^ (2^−△Ct^ mean±SE)	0.3153±0.0604*	0.3396±0.0576*	0.1216±0.0183
GSTM2^a^ (2^−△Ct^ mean±SE)	0.5784±0.1210*	0.3927±0.0897	0.1433±0.0246
GSTA3^b^ (2^−△Ct^ median)	0.0057*	0.0048	0.0024
SOD2^b^ (2^−△Ct^ median)	0.094	0.0821	0.0805
GSTP1^b^ (2^−△Ct^ median)	0.0562	0.049	0.0268
HMOX1^b^ (2^−△Ct^ median)	0.0093	0.0093	0.0073
NRF2^b^ (2^−△Ct^ median)	0.2749	0.2413	0.1339
GCLM b (2^−△Ct^ median)	0.0766	0.0712	0.0538
GCLC b (2^−△Ct^ median)	0.0291	0.0324	0.0209

CSE: cigarette smoke extract. SOD2: superoxide dismutase. GSTP1: glutathione-S-transferase P1. HMOX1: heme oxygenase-1. NRF2: nuclear factor erythroid 2-related factor 2. GSTM1: glutathione S-transferase Mu 1. GSTM2: glutathione S-transferase Mu 2. GSTA3: glutathione S-transferase alpha3. GCLM: glutamate cysteine ligase modifier subunit. GCLC: glutamate cysteine ligase catalytic subunit.

a The data was normally distributed (P>0.05, Shapiro-Wilk), with unequal variances (P<0.05, ANOVA), and was analysed with Tamhane's T2 test.

b The data from at least one group was not normally distributed (P<0.05, Shapiro-Wilk test).

*<0.05 versus menadione.

GSTM1 expression in the CSE group was insignificantly higher than in the control group (CSE 0.3396±0.0576, control 0.3153±0.0604, P = 0.989, ANOVA *post-hoc* Tamhane's T2 test); in contrast, there was a significant diminution in the expression in the menadione group compared to the control group (menadione 0.1216±0.0183, control 0.3153±0.0604, P = 0.044, ANOVA *post-hoc* Tamhane's T2 test), there was a similar significant difference between the CSE and menadione groups (CSE 0.3396±0.0576, menadione 0.1216±0.0183, P = 0.025, ANOVA *post-hoc* Tamhane's T2 test) (Table 3, Dataset S8).

GSTA3 expression in ovaries from the CSE group was lower than that in the control group (CSE 0.0048, control 0.0057, P = 0.271, Mann-Whitney test), and that of the menadione group was also changed (CSE 0.0048, menadione 0.0024, P = 0.141, Mann-Whitney test). Although neither of these two changes was significant, there was a significant decrease in expression in the menadione group compared with the control group (menadione 0.0024, control 0.0057, P = 0.049, Mann-Whitney test) (Table 3, Dataset S14). Expression of all other genes was unaffected by treatments (Table 3, Dataset S7, S10-S13, S15-S16).

## Discussion

From our study we have concluded that exposure to CSE alters several reproductive parameters in mice: a reduction in the ZP-free oocyte size and the number of ARS-PB; more SSR-PB.

According to our data, a positive control (menadione) was successfully established with a significantly lower level of GSTM1, GSTM2 and GSTA3, a thinner ZP, larger PVS, and higher rate of immature oocyte.

Tobacco smoke as a source of exogenous pro-oxidants, such as ROS and free radical generators, can cause oxidative damage [49]–[51], and smoking may increase ROS and the depletion of redox scavengers in peripheral blood [49], [52]. Shifting the dynamic balance between pro-oxidation and antioxidation can lead to oxidative stress [53], [54]. Active smoking affects the pro-oxidant/antioxidant balance inside the pre-ovulatory ovarian follicle in women undergoing ovulation induction for IVF [55]. Siddique [56] assessed the influence of cigarette smoke condensate (CSC) and B[a]p on the levels of oxidative stress biomarkers, in *in-vitro* spent media of follicle cells and concluded that CSC and B[a]p exposure could induce oxidative stress in ovarian follicles. Similarly, Gannon [29] reported that exposure to smoke components caused oxidative stress with increased heat shock protein 25 (Hsp25), a kind of stress protein, and decreased SOD activities. In our study, SOD2 expression had a slight decline in CSE just like the positive control group, though non-significantly. In addition, similar to the remarkable decrease in the menadione positive control group, the expression of GSTM2 and GSTA3 in CSE exposure ovary had a mildly reduced level compared with control group. GSTs, a super-gene family composed of multifunctional enzyme systems [57], [58], catalyze the conjunction between intermediate metabolites of xenobiotic metabolism and GSH. The conjugates gain a reduced toxicity and are then easy to be expelled. This process exerts an important effect on the cellular detoxification of electrophilic compounds and the antioxidative reactions protecting lipids [40]. Both GSTM2, a cell-type GSTs, and GSTA3, belonging to the α class GSTs [42], [44], are involved in antioxidative reactions. Lim [40] demonstrated that the expression of GSTM2 in the ovary may be significant in protecting oocytes from toxic substances and the decrease in mRNA expression of the cytosolic antioxidant GSTM2 is involved in ovarian oxidative damage to lipids, proteins, DNA, and other cellular components vital for maintaining ovarian function and fertility.

The effects of cigarette smoke on oxidative stress are well known as are the effects of smoke on cellular apoptosis [59]–[61]. Increased lipid peroxidation, reduced glutathione contents, increased catalase activity, decreased SOD activity, cytoplasmic retractions and fewer intercellular junctions were observed in granulosa cells exposed to Cd [62], [63], a heavy metal compound in cigarette smoke. *In vitro*, B[a]P was shown to inhibit gap junction formation [64], and junctions being indispensable for oocyte-granulosa cell cross-talk [65]. So we can hypothesis that cigarette smoke may have detrimental effects on oocyte through inducing oxidative stress and injuring granulosa cells.

The studies about the effect of smoke on oocyte are few. Smokers present a lower estradiol (E_2_) level during ovarian stimulation in IVF [66], [67]. Inhibition of follicle growth and decreased E_2_ synthesis were demonstrated [13], [68], which was associated with the oocyte of poor quality. Sobinoff's study [69] concluded that B[a]P exposure caused mitochondrial leakage resulting in reduced oolemma fluidity and impaired fertilization in adulthood, resulting in oocyte aging and dysfunction, which was supported by Gruber's finding [70]. In our study, OD in the CSE group was non-significantly less than that in the control group, in contrast to the positive control (menadione); and there was a notable reduction in the ZP-free OD in the CSE group compared with the control group. Similarly, a smaller OD in incipient antral follicles was found in mice after nicotine exposure and ex-smoking mice showed an increase in OD compared to smoking mice [4]. Some researchers have concluded that OD was clearly relevant to meiotic maturation and the developmental potential exhibited by embryos after *in-vitro* maturation, IVF, or *in-vitro* culture [71]. The reduction in oocyte size has been widely accepted to be one of the apparent characteristics of apoptosis [72]. Some investigators considered that it was related to Bax gene expression and oocyte destruction mediated by PAH correlated with activation of relevant genes governing programmed cell death (PCD) [73], [74]. There are also several studies about the effect of smoke on oocytes morphology, such as thicker ZP [30], [31], leading to difficult fertilization, though the alteration in our study was not significant.

Investigators have considered the morphology of PB to be one of the indices to be used for evaluation of overall oocyte viability [75] and an indicator of aging in ovulated oocytes [76]. However, studies on the relationship between cigarette smoke and first PB morphology are few. We demonstrated that the CSE group showed a noticeably lower rate of ARS-PB and a higher rate of SSR-PB than the control group, and exhibited a stronger effect than that observed in menadione group. Additionally, the incidence of broken PB after CSE exposure was greater, although it was not statistically significant. It was considered that the oocytes with smooth and intact PB are expected to engender a higher fertilization rate and better embryo quality [76]; and this type of PB has been correlated with an increase in development to the blastocyst stage and overall pregnancy rate [77], [78]. According to our study, though ZP thickness, PVS, immature oocyte rate and the rate of broken PB were non-significantly altered, we can conclude that following CSE exposure, the mouse oocyte is affected negatively.

Synthesizing all the researches mentioned above, it's reasonable to suppose that cigarette smoking may potentially emerge a lower rate of fertility and successful pregnancy, producing oocyte of poor quality, through oxidative stress. For further study, we will perform *in-vitro* fertilization or intracytoplasmic sperm injection (ICSI) in each group to prove this standpoint in the future.

In the research from Whitcomb [79], compared with non-smokers, smokers had higher levels of follicle-stimulating hormone (FSH) in the early follicular phase (7.9 mIU/mL versus 6.3 mIU/mL) after adjusting for potential confounding factors, such as age, similar to that from Cooper [80]. Freour [81] found that anti-Mullerian hormone (AMH) was significantly lower in smokers (3.06 versus 3.81 mg/l). Higher FSH and lower AMH, we know that, were associated with lower reserve and aging of ovary. Many studies involving IVF procedures provided evidence that cigarette smoke had deleterious effects on ovaries: lower sensitivity [82], [83] and fewer retrieved oocytes [67], [84]-[86]. The number of ovulation of our study had no statistically significant alteration, similar to the data from other researchers [19], [33]. Maybe more thorough and large studies are needed for a consistent consequence.

Combined with many evidences that smoke and its component causing follicle depletion [25]-[29] and the inhibition of follicle growth [13], it is reasonable to suppose that cigarette smoke may do harm to ovary, causing impaired ovary function, fewer follicles, oocyte of poor quality, through inducing oxidative stress.

## Conclusion

According to our study, we suggested that CSE exposure was associated with a shrink size and poor morphology of oocytes and oxidative stress maybe the underlying mechanism. We certainly recommend that quitting smoking is a wise choice to ensure good fertility.

## Supporting Information

Dataset S1 **Data of ovulation quantity.**
(SAV)

Dataset S2 **Data of ZP thickness.**
(SAV)

Dataset S3 **Data of ZP-free OD.**
(SAV)

Dataset S4 **Data of PVS.**
(SAV)

Dataset S5 **Data of OD.**
(SAV)

Dataset S6 **Data of PB.**
(XLS)

Dataset S7 **Ct of 3 replication of ACTIN.**
(SAV)

Dataset S8 **Ct of 3 replication of GSTM1.**
(SAV)

Dataset S9 **Ct of 3 replication of GSTM2.**
(SAV)

Dataset S10 **Ct of 3 replication of GSTP1.**
(SAV)

Dataset S11 **Ct of 3 replication of HMOX1.**
(SAV)

Dataset S12 **Ct of 3 replication of NRF2.**
(SAV)

Dataset S13 **Ct of 3 replication of SOD2.**
(SAV)

Dataset S14 **Ct of 3 replication of GSTA3.**
(SAV)

Dataset S15 **Ct of 3 replication of GCLC.**
(SAV)

Dataset S16 **Ct of 3 replication of GCLM.**
(SAV)
